# The Murine Epithelial Thymic Microenvironment Is Governed by Eph/Ephrin Signalling

**DOI:** 10.3390/cells15181638

**Published:** 2026-09-10

**Authors:** Sara Montero-Herradón, David Alfaro, Agustín G. Zapata, Javier García-Ceca

**Affiliations:** 1 Department of Cell Biology and Histology, Faculty of Biological Sciences, Complutense University of Madrid, 28040 Madrid, Spain; saramontero@ucm.es (S.M.-H.); david.alfaro@bio.ucm.es (D.A.); zapata@ucm.es (A.G.Z.); 2 Health Research Institute, Hospital 12 de Octubre (imas12), 28041 Madrid, Spain

**Keywords:** thymus, thymic epithelial cells, thymocytes, Eph/ephrinB, EphA

## Abstract

**Highlights:**

**What are the main findings?**

- The absence of Eph receptors or their ligands, the ephrins, results in specific and profound alterations in the thymic epithelial stroma.
- These alterations in EphB2- and/or EphB3-deficient thymuses are associated with hypocellularity and slight changes in T cell differentiation but do not result in altered proportions of peripheral T lymphocytes.

**What are the implications of the main findings?**

- These findings partially question the importance of thymic epithelial cell–thymocyte interactions in the thymic functions.
- This suggests that just a certain number of thymic epithelial cell–thymocyte interactions might be sufficient to provide functional T lymphocytes.

**Abstract:**

Although the relevance of thymic epithelial cell–thymocyte interactions for thymus function has long been emphasised, the mechanisms that govern the biology of thymic epithelial cells (TECs) are not fully understood. In other epithelial tissues, erythropoietin-producing hepatocellular carcinoma (Eph) receptors and their ligands, the Eph receptor-interacting proteins (ephrins), have been demonstrated to be important regulators of epithelial homeostasis. Accordingly, we have studied the function of Eph/ephrin signalling in the organisation, phenotypes and function of mouse thymic epithelial cells, as well as its effects on T-cell differentiation, for over 20 years. This review provides a detailed description of these results, concluding that the absence of certain EphA/B receptors or ephrins B generates specific phenotypes that affect T-cell differentiation and immunological function in different ways. This suggests that the proper histological organisation of the epithelial thymic stroma is not essential for normal T-cell maturation, or that at least some aspects of the process are preserved.

## 1. Introduction

The thymus is a primary lymphoid organ that plays a pivotal role in the maturation and immune education of T cells. It ensures an efficient response to foreign antigens, while also preventing self-aggressive immune reactions. For this reason, it is considered a crucial component of central immune tolerance.

Considerable attention has been paid to understanding the molecular and cellular mechanisms that govern the functional maturation of thymocytes, particularly focusing on the importance of cell interactions between T cells and thymic epithelial cells (TECs). Nevertheless, the homeostasis of the thymic stroma is still not well understood. We [1,2] and other authors [3,4,5] have identified the role of erythropoietin-producing hepatocellular carcinoma (Eph) receptors, which are the most abundant tyrosine kinase receptors in animal cells, and their ligands, Eph receptor-interacting proteins (ephrins), in regulating epithelial tissues, including the thymus.

The following sections of this review will demonstrate that Eph and ephrin B, which are expressed in both developing thymocytes and TECs, play a role in the colonisation of lymphoid progenitors in the thymus and the migration of developing thymocytes throughout the thymic parenchyma. This ensures the correct topological location of distinct T cell subsets, which receive environmental instructions largely from different TEC subpopulations. Furthermore, EphB2 and EphB3 play an autonomous role in regulating the progression of double-negative (DN, CD4^−^CD8^−^) cells to the double-positive (DP, CD4^+^CD8^+^) cell compartment, and DP cells to single-positive (SP, CD4^+^CD8^−^ and CD4^−^CD8^+^) T cells, by controlling interactions between thymocytes and TECs. Similarly, altered maturation of thymocytes affects the establishment of the thymic epithelial network and the emergence of distinct TEC subsets. Additionally, distinct phenotypes found in other Eph- or ephrin-deficient thymuses confirm the specificity of these signalling pathways.

## 2. Expression of Eph and Ephrins of Families A and B in the Thymus

Eph and ephrin molecules are categorised into two families: EphA (nine members in mammals: A1–A8 and A10) and EphB (five members: B1–B4 and B6). This classification is based on the similarities within each group of the extracellular domains of the molecules, as well as their affinity for the ligands, ephrins. Ephrins A are glycophosphatydilinositol-anchored proteins that are found on the cell surface (five members, A1–A5), while ephrins B (three members, B1–B3) possess a single transmembrane domain [6]. Each Eph receptor can bind to several ephrins and vice versa; exceptionally, a few Ephs (e.g., EphA4) interact with both ephrins A and B [7].

Most Eph receptors are active tyrosine kinases, whereas ephrins lack intrinsic catalytic activity, but they bind to several intracellular targets and are associated with tyrosine kinases. Accordingly, stimulation of Eph/ephrin pairs results in bidirectional signalling in both Eph- (forward signals) and ephrin-expressing cells (reverse signals), as well as cell- and non-cell autonomous responses [8,9].

Pioneering studies of the rat thymus have reported that ephrins A are principally expressed in thymocytes, whereas Eph expression is restricted to TECs. However, both EphA1 and ephrinA1 are present in both cell types [10]. Furthermore, the same cell type or thymic compartment often expresses multiple Ephs and ephrins [10,11]. Indeed, all thymocyte subsets, as well as cortical TEC (cTEC) and medullary TEC (mTEC) subsets, express Eph and ephrins, particularly those of the B family [11,12]. During the early stages of thymic development, when TEC numbers exceed those of thymocytes, the proportions of cells expressing EphB2, EphB3 and ephrinB2 reach their highest values. These values decrease sharply later on, except for ephrinB2, which remains high until embryonic day (E) 14.5. Consequently, the proportion of TECs expressing Eph and ephrin B is higher than that of Eph/ephrin-positive thymocytes. This suggests that the thymic epithelium would be subject to more severe effects in the absence of Eph/ephrinB signalling in the early stages of development [12].

The capacity for bidirectional signalling, together with the promiscuous interactions established between several Ephs and ephrins, determines a high number of distinct cell interactions that enable these molecules to be involved in a wide range of cellular functions. These include cell attachment/detachment, migration/chemotaxis, topological positioning, cellular shape, the regulation of gene expression, cell death, proliferation, and differentiation [8].

## 3. The Lack of Eph or Ephrins Courses with Differentially Altered Development of Both the Thymic Epithelium and the Thymocytes

### 3.1. Thymuses Deficient in Eph or Ephrins Exhibit Extremely Low Numbers of Cells

Our first experiments revealed in vitro the importance of Eph receptors for the structure and function of the murine thymus. These experiments demonstrated that EphA-Fc fusion proteins inhibited the differentiation of rat thymocytes, leading to a reduction in their numbers (mainly DP cells) and an increase in the proportion of apoptotic cells.

Later, we studied the thymuses of different Eph-deficient mice, kindly provided by Dr Charnay (L’ Institute National de la Santé et de la Recherche Medicale, École Normale Superiere, Paris, France) (EphA4^−/−^ mice) and Dr Henkemeyer (University of Texas, Southwastern Medical Center, Dallas, Texas, USA) (EphB2^−/−^ and/or EphB3^−/−^ mice). In general, a lack of Eph or ephrin A or B signalling results in increased apoptosis of DN and DP T cells [11], as well as TECs [13]. This is often, but not always, accompanied by a reduction in the proportion of cycling cells [14]. However, other studies have reported no thymus phenotype in mice deficient in EphB2 [15] or EphB6 [16,17], suggesting that the absence of any Eph or ephrin signalling is compensated for by signals mediated through other molecules in these families. Nevertheless, EphB6 overexpression under CD2 promoter control is associated with breakdown of the thymic cortex/medulla limits, hypocellularity, and increased proportions of DN cells [15]. More recent studies have also reported significant changes in the EphB2-deficient thymus [11,14]. Indeed, studies on the phenotypes of mutants deficient in different Ephs or ephrins reveal specific features for each mutant, which will be described in more detail later.

Regardless of the observed thymus phenotype, a lack of Eph/ephrin signalling leads to a profound hypocellularity, reflecting changes in the two main thymic compartments, thymocytes and TECs, and their Eph/ephrin-mediated cell interactions. In vitro, foetal thymus organ cultures (FTOCs) supplied with EphA1-Fc, EphA2-Fc, EphA3-Fc or ephrinA1-Fc fusion proteins exhibited reduced absolute cell numbers, with the latter condition being particularly severe because ephrinA1-Fc proteins presumably blocked most EphA signalling [10]. These treated lobes contained increased proportions of apoptotic cells, primarily in the immature CD4^−^CD8^+^ cell and DP thymocyte compartments. However, there were no changes in the proportions of cycling cells. Similar results were observed in FTOCs receiving soluble EphB2-Fc or ephrinB1-Fc fusion proteins [18]. However, other studies have reported that treatment of FTOCs with ephrinB1-Fc, but not ephrinB2-Fc or ephrinB3-Fc fusion proteins, decreases cell content [19].

On the contrary, some data suggest that activation of the Eph/ephrin pathway reduces apoptosis levels [19,20,21]. Nevertheless, crosslinking of EphB6 induces apoptosis in Jurkat cells [22], and both immobilised EphB2-Fc and ephrinB1-Fc proteins modulate anti-CD3 antibody-induced apoptosis in DP thymocytes, as well as the formation of conjugates between DP thymocytes and wild-type (WT) TECs [18]. To better understand these controversial results, it is important to note that the effects of the Eph/ephrin signalling presumably depend on the density of the immobilised proteins on the plate surface [18].

Another factor that could contribute to the hypocellularity of EphB-deficient thymuses is whether they are properly (or not) seeded by lymphoid progenitor cells, given that they have no self-renewing lymphoid progenitor cells. Two factors are important for this colonisation: the number of lymphoid progenitor cells that can colonise the organ, and the efficiency of the mechanisms involved in their migration.

Results from the bone marrow (BM) haematopoietic progenitor cells of EphB-deficient mice demonstrated a decreased proportion of early haematopoietic cells in EphB2^−/−^ BM [23]. However, the role of these cells in thymus seeding remains unclear [24]. Conversely, neither foetal liver CD45^+^PIRA/B^+^ precursors [25] nor later BM progenitor cells expressing molecules known to be involved in thymus seeding (e.g., CCR7, CCR9, CXCR4 and PSGL1) showed significant changes in the analysed mutant mice [23]. Nevertheless, in vivo and in vitro assays have shown that a lack of EphB in either lymphoid progenitors or thymic stroma significantly reduces thymic seeding in both foetal [26] and adult [23] mice. Taken together, these results suggest that migration mechanisms are more important than the number of available lymphoid precursors. To confirm this hypothesis, we analysed possible changes in the expression of some molecules such as chemokines (CCL25, CCL21 and CXCL12), extracellular matrix (ECM) components (fibronectin (FN) and laminin (LN)) and their receptors (CCR9, CCR7, CXCR4, and VLA-4, VLA-5 and VLA-6, respectively), which are known to be involved in lymphoid progenitor seeding in the thymus and/or the migration and specific positioning of developing thymocytes throughout the thymic stroma.

Thus, foetal EphB2- and EphB3-deficient thymuses exhibited reduced expression of not only the chemokines CCL21 and CCL25 at E12.5 and E13.5 but also CXCL12 at E13.5 [25]. Conversely, adult EphB2-deficient mice exhibited decreased production of chemokines, FN and LN. However, there were no changes in integrin and chemokine receptor expression [26]. Additionally, transwell-based experiments revealed that Lin^−^Sca1^+^c-Kit^+^ (LSK) progenitor cells lacking EphB2 or expressing EphB2LacZ (a truncated form of EphB2 that cannot transmit forward signals) exhibited decreased transmigratory responses compared to WT cells in the presence of any stimulus. Furthermore, Eph stimulation by coated ephrinB1-Fc fusion proteins also caused reduced migration of WT BM progenitor cells [26]. Other authors have emphasised the importance of Eph/ephrins in the migration of peripheral immune cells. For instance, ephrinB1 stimulation inhibits chemokine-induced lymphocyte migration [27], whereas ephrinB1-Fc fusion proteins stimulate the CXCL12-dependent migration of peripheral blood lymphocytes [28].

Furthermore, P-selectin, which is involved in progenitor cell migration into the adult thymus [29], showed reduced expression in the endothelial cells of both EphB2- and EphB3-deficient thymuses, but not EphB2LacZ ones [23]. Decreased migration in EphB2^−/−^ thymuses also correlates with reduced expression of ephrinB1 and ephrinB2. In EphB3^−/−^thymuses, the reduction only affected ephrinB1 expression, which supports the idea that forward signals mediated by the EphB2/ephrinB1 pair are especially important for intrathymic lymphoid migration [23].

Although thymus seeding is relevant to the hypocellularity of mutant thymuses, increased apoptosis of both thymocytes and TECs appears to be an even more important factor. Whereas CCR9/CCR7 double knock out (KO) mice undergo profound depletion of early T cell progenitors, they show a recovery in later stages, possibly through compensatory proliferation of some DN2 (CD44^+^CD25^+^ DN) and DN3 (CD44^−^CD25^+^ DN) precursor cells [30,31]. However, there is no compensatory proliferation in EphB2-deficient mice, which exhibit severely reduced cell numbers from the DN3 cell stage onwards, in correlation with increased apoptosis, primarily of DN and DP cells, and decreased proportions of DN cells [11,25].

Taken together, all these factors, low numbers and reduced maturation of seeding lymphoid progenitors [11,18,25], increased apoptosis, and reduced proportions of cycling thymocytes [11], could be due to decreased numbers of Dll4 [12,32] and IL7 receptor α chain transcripts [33]. These two molecules, which are both involved in DN-DP cell progression [32,34], also contribute to the hypocellularity of mutant thymuses [12].

Reduced foetal lymphoid immigration affects the proportion of immature MTS-20^+^ TECs [25], which, in turn, leads to a decrease in total TEC numbers. Accordingly, altered proportions of cycling and apoptotic TECs also account for thymic hypocellularity, which affects the proper development of the thymic epithelium. Thus, a high proportion of apoptotic TECs correlates with a reduced K8^+^ epithelial network [13] and low TEC proliferation at E12.5. This is a consequence of the delayed seeding of lymphoid progenitors [12], resulting in a decreased proportion of cycling TECs. This could be partially related to reduced transcripts of both FGF7 and its receptor, FGFR2IIIb [12], both of which are involved in TEC proliferation [35,36]. Interestingly, Eph/ephrinB signalling activates FGFR2IIIb in other systems [37], so its absence in EphB-deficient mice could explain the decrease in FGFR2IIIb transcripts. Delayed maturation of the mutant thymuses was confirmed by analysing distinct TEC subsets by flow cytometry (see later). These changes culminated in an adult phenotype characterised by a disrupted three-dimensional (3D) epithelial network due to the inability of thymocytes and TECs to intermingle adequately.

### 3.2. A Lack of EphB2, EphB3, EphrinB1 or EphrinB2 Leads to Changes in TEC Maturation and the 3D Organisation of the Epithelial Network

These alterations include the accumulation of K5^+^K8^+^ thymic epithelial precursor cells in the cortex and the occurrence of K5^−^K8^−^ epithelial-free areas (EFAs). Numerous epithelial cysts also appear, which are scarcely represented in WT murine thymuses [14,38]. Additionally, increased proportions of apoptotic TECs are evident in these mutants, primarily comprising immature EpCAM^+^MTS20^+^ cells and EpCAM^+^Ly51^+^ cTECs [13]. Similarly, in vitro activation of either EphB or ephrinB signalling decreases the frequency of apoptosis in WT TECs, whereas its elimination increases apoptosis. Interestingly, reaggregate thymic organ cultures (RTOCs) formed exclusively with EphB-defective TECs yielded a higher proportion of apoptotic cells than RTOCs consisting of TECs and total thymocytes, suggesting the relevance of Eph/ephrin-mediated thymocyte–TEC interactions for TEC survival [13].

**Figure 1:**
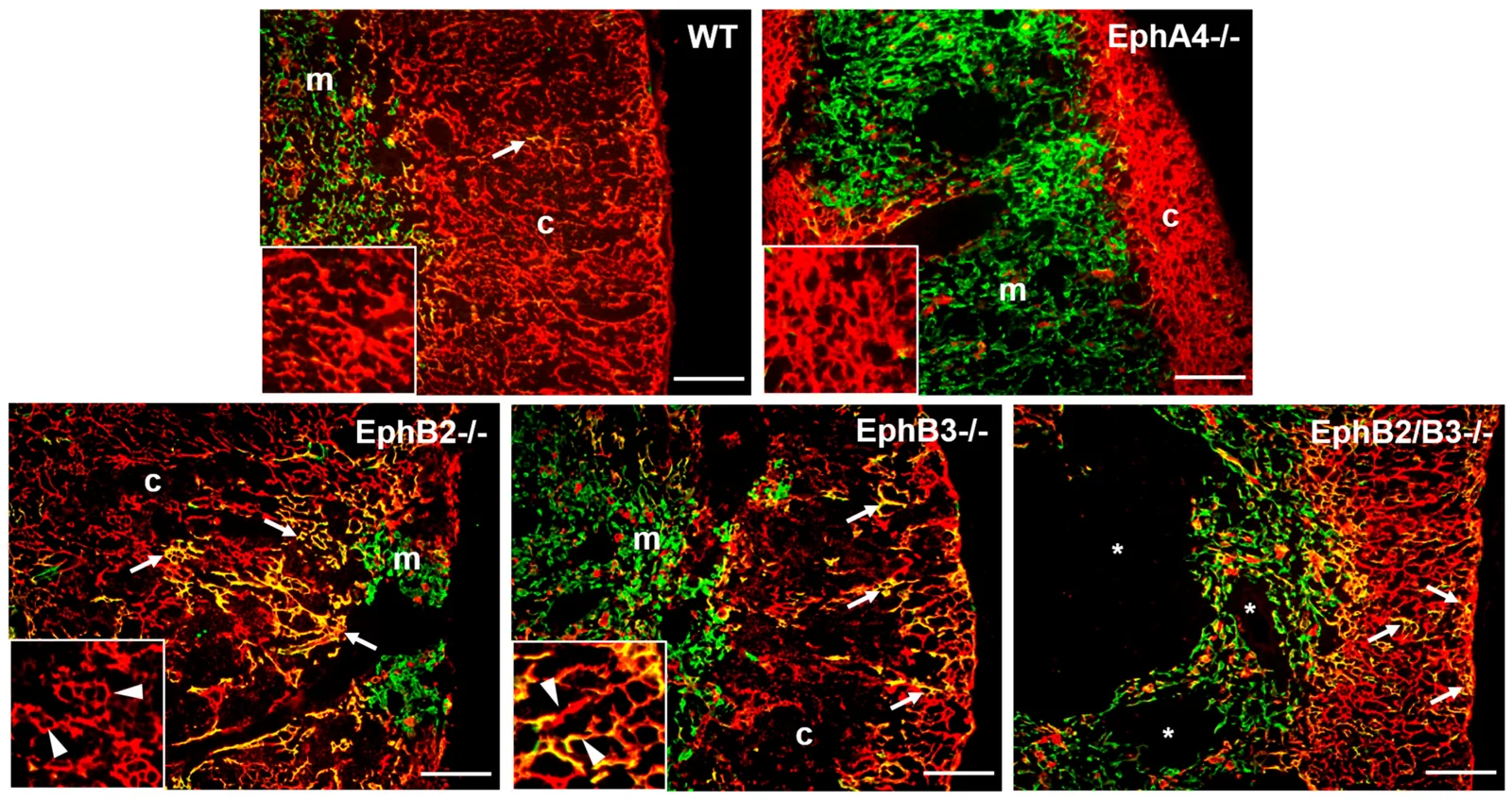
Representative immunofluorescence analysis of thymic epithelial cell network organisation in WT and Eph mutant thymuses. The images illustrate the main features of these thymuses. WT thymuses exhibit a well-organised outer cortex (K5^−^K8^+^, c), which is densely populated by epithelial cells (insert), and a single inner medulla (K5^+^K8^−^, m). EphA4^−/−^ thymuses, which contain a reduced number of CD4^+^CD8^+^ thymocytes, are characterised by a large inner medulla that correlates with a smaller, densely packed cortical area (insert). EphB2^−/−^ thymuses exhibit cortical regions with few epithelial cells and scattered medullary areas. Cortical TECs generally have shortened epithelial cell processes and acquire a rounded morphology (insert, arrowhead). EphB3^−/−^ thymuses contain an inner medulla, but the cortical area is formed by a reduced number of TECs with elongated, perpendicular processes extending from the thymic capsule (insert, arrowhead). The medulla of EphB2/B3^−/−^ thymuses contains large areas of free epithelial tissue (*). All EphB-mutant thymuses exhibit an increased proportion of immature K5^+^K8^+^ cells in the cortical area (arrows). Thymic sections were obtained from 8-week-old EphB-deficient female mice and 3-week-old EphA4-deficient female mice, all of which were on a CD1 background. K5: green; K8: red. Scale bar: 100 μm.

It is important to note that the studied EphB- or ephrinB-deficient mice are phenotypically different. Therefore, the lack of one receptor is not compensated for by the presence of another. Despite the fact that the absence of such receptors has been associated with compensation by other expressed Ephs or ephrins, the phenotypes are not overlapping, but rather specific and non-redundant. The thymuses of both EphB3^−/−^ and EphB2/B3^−/−^ mice contain a large central medulla, whereas in those deficient in EphB2, the thymic medulla is reduced to small areas scattered throughout the parenchyme (Figure 1). The cortex is also disorganised, exhibiting increased expression of the medullary marker K5, and the cTECs have lost their typical perpendicular disposition with respect to the connective tissue capsule. The EphB3^−/−^ cortex is less disorganised, maintaining parallel columns of TECs with long cell processes and a high number of K5^+^K8^+^ cells. In contrast, EphB2^−/−^ TECs exhibit shortened cell processes [14] (Figure 1). EphB2/B3^−/−^ double mutants exhibit characteristics of both single-defective mice, including some severely disorganised areas alongside regions consisting of cells with long cell processes. Ephs and ephrins regulate the emission and arrangement of cell processes in other cell types, such as neuronal dendritic spines [39] and integrin-mediated cell interactions with ECM components [40,41]. Additionally, we have demonstrated that ephrinB1-Fc fusion proteins supplied to RTOCs formed by WT TECs and DP thymocytes induce retraction of the epithelial cell processes [11].

Once again, K5^+^K8^−^MTS10^+^ cells and K5^+^K8^+^MTS10^+^ mTECs are present in the cortical area. An increased number of K5^+^K8^+^ cells is not related to the high number of MTS-20^+^ thymic epithelial progenitor cells. However, it is apparently linked to K5^+^K8^+^MTS-20^−^ cells, found in EphB-deficient thymuses, and may be related to altered regulation of the K5 expression in TECs. The increased number of rare K5^−^K8^−^MTS-20^+^ cells observed in these mutants may be due to downregulation of K5 and K8 expression in MTS-20^+^ epithelial progenitor cells [14]. Conversely, a high number of K5^+^K8^+^ cells has been reported in other mutants with defects in molecules involved in TEC development, such as Foxn1^∆/∆^ [42], conditioned Stat3-deficient [43] and Kremen1^−/−^ mice [44]. These molecules, including EphB, are associated with Stat3 activation [45] and indirectly affect the activity of the transcription factor AP-1 [46], which regulates the keratin gene promoter [47]. Furthermore, adult thymuses from both hCD3ɛTg26 and Rag2^−/−^γc^−/−^, which exhibit a significant T cell differentiation blockade similar to EphB-deficient thymuses, also exhibit increased numbers of K5^+^K8^+^ TECs [48].

Clearly, these phenotypes suggest that TECs from EphB-deficient thymuses are physically separated from other TECs and thymocytes. Nevertheless, they still express functional markers, such as MHCII molecules [14,49]. While EphB2 and EphB3 are both necessary for the proper development of the cortical and medullary thymic epithelium, a lack of EphB2 results in a more severe medullary phenotype than a lack of EphB3. In contrast, the thymic cortex is particularly affected in EphB3^−/−^ mice [12,49]. A lack of EphB, particularly EphB3, results in delayed cTEC maturation from the earliest stages of development [12]. Thus, at E14.5/15.5, mutant thymuses accumulate immature Ly51^+^CD205^−^ cTECs, while the proportion of mature Ly51^+^CD205^+^ cells decreases. Other cTEC subsets identified by different markers, such as CD40, MHCII and β5t, exhibit a similar developmental pattern. Changes in the thymic medulla are more evident in EphB2^−/−^ thymuses, with an accumulation of Claudin3,4^hi^SSEA-1^+^ thymic epithelial precursor cells, a reduction in mature mTECs defined by the expression of UEA-1, MHCII, CD40, CD80 and Aire, and reduced expansion of medullary islets [49]. Remarkably, E13.5 alymphoid foetal EphB2^−/−^ thymus lobes grafted under the kidney capsule of WT mice showed a more fragmented medulla compared to those derived from EphB3-deficient thymuses [49].

The changes undergone by the mTECs of EphB-deficient mice may be mediated by molecules in the TNF/TNFR superfamily, particularly by the RANK/RANKL pair [50]. The absence of this pair has a profound effect on Aire^+^ mTEC subsets [51,52]. Other molecules that are presumably involved in this regulation include osteoprotegerin [51] and LTβR [53]. To confirm this, we stimulated the RANK/RANKL pair to recover the defective maturation of mutant mTECs. EphB-deficient E14.5 alymphoid thymic lobes treated with an agonist anti-RANK antibody restored the WT proportion of mature CD40^hi^CD80^hi^ cells and MHCII^+^UEA-1^+^ mTECs. Conversely, mutant thymuses showed a significant reduction in the proportion of E15.5 Vγ5^+^RANKL^+^ cells, which presumably resulted in a weak RANK signal in mTECs [49].

Large areas devoid of epithelial cell marker expression, of unknown significance, are frequently found in EphB-deficient thymuses and are known as EFAs (Figure 1). These areas also occur in WT thymuses of different species, such as rats [54], mice [55], and humans [56]. EFAs are particularly evident in EphB2/B3 double KO mice [14,57] and, remarkably, in mutant mice with defects in molecules involved in TEC maturation, such as Foxn1, Kremen1 and Stat 3 [42,43,44]. In these areas, thymocytes and blood vessels are often surrounded by enlarged sheaths of connective tissue. In EphB2^−/−^ thymuses, EFAs appear scattered throughout the thymic parenchyma, whereas in EphB3^−/−^ thymuses, they are largely confined to the medulla and inner cortex. In double EphB2/B3 mutants, however, EFAs are large, “emptied” areas that spread from the medulla into the cortex (Figure 1) [14,57].

EFAs are distinct in the thymic cortex and medulla. The former contains thymocytes and some sheathed blood vessels, whereas the medulla contains mTECs that delineate areas with enlarged blood vessels, increased numbers of ER-TR7^+^ fibroblasts, ECM components (collagen type IV, FN and LN) [58], dendritic cells [59], and, in some areas, thymocytes [60]. These cortical areas have been described as MHCII-negative and poorly vascularised areas containing abundant thymocytes, which are frequently in division [60]. They are considered accumulations of DP thymocytes that do not undergo positive selection and subsequently undergo apoptosis [61]. On the contrary, medullary EFAs that express several connective tissue markers may be in a mesenchymal condition [58] and have undergone epithelial–mesenchymal transition, losing epithelial cell markers and acquiring a mesenchymal condition. However, we did not demonstrate the expression of markers associated with epithelial–mesenchymal transition in the medullary EFA, including Snail, Slug, FSP1 and vimentin [57]. Furthermore, the abundance of EFAs in EphB mutants may be a consequence of impeded intermingling and mutual exclusion of thymocytes and TECs due to the absence of Eph/ephrin signalling, as observed in other systems [62,63].

The possible functions of EFAs are largely unknown. It has been suggested that EFAs are responsible for generating auto-reactive cells [64]. However, Wistar rats with large EFAs exhibit a very low incidence of autoimmune diseases [60], and no immunological abnormalities (see later) have been reported in the proportions of T cell subsets defined by CD4/CD8 marker expression in EphB-deficient mice [1]. Accordingly, we propose that these areas have no functional immunological significance per se, but merely reflect a lack of intermingling between thymic cell components, as well as an increased proportion of apoptotic TECs, as a consequence of altered thymic cell interactions. Nevertheless, we cannot rule out the possibility that EFAs are related to other processes in other models.

On the other hand, selective deletion of ephrinB1 and/or ephrinB2 genes in either thymocytes or TECs has provided valuable insights into the organisation and compartmentalisation of the thymic epithelial microenvironment [38,65]. The specific absence of ephrinB1 and ephrinB2 on either thymocytes or TECs results in more severe and specific changes to the thymic epithelium, affecting cTEC subset phenotypes and location, thymus size, and the organisation of the medullary epithelium [38,65]. Deletion of ephrins in immature thymocytes induces hypocellularity and a partial blockade of T-cell maturation, significantly impacting the DN cell compartment and increasing the proportion of DN3 cells. Remarkably, our results show that ephrinB1 and/or ephrinB2 deficiencies in TECs affect thymocyte positive selection. These thymuses have lower proportions of TCRαβ^hi^CD69^hi^ cells within the DP cell subset [65]. However, it should be noted that, as has been reported for other Ephs and/or ephrins [66,67,68], the genetic background contributes to determining the severity of the phenotypes. The most severe and variable phenotypes occurred in C57BL/6-CD1 mixed-background mutant mice rather than in the C57BL/6 strain mice [65].

Two subsets of cTECs have been defined based on Ly51 expression. It has been concluded that Ly51^hi^ cTECs would basically correspond to the niche of DN thymocytes, while Ly51^lo^ cTECs would house DP thymocytes [69]. However, some ephrinB-deficient mutants, in which the relevant molecules were deleted in the thymocytes, showed more homogeneous Ly51 expression throughout the mutant thymus, making it impossible to distinguish between Ly51^hi^ cells and Ly51^lo^ cells. Furthermore, cTECs tended to form rounded groups with thymocytes rather than being radially arranged [65]. Analysis of the mutant thymuses in which the ephrinB1 and/or ephrinB2 genes were specifically deleted in TECs revealed a delay in the sequential appearance of cTECs subpopulations, with no change in the proportion of MTS-20^+^ cTECs within each Ly51^+^ cell subset compared to WT values. In contrast, most of the mutants studied showed increased proportions of K5^+^K8^+^ TECs, some of which were Ly51^hi^K5^+^ cells.

EphrinB1 and ephrinB2 also play important roles in the organisation of the thymic medulla [38]. In all mutants studied, the medullary areas were smaller and more compact than those in WT thymuses. Additionally, large epithelial cysts and increased proportions of UEA-1^hi^MTS-10^−^ cells were frequently observed compared to the WT medulla. In mutant thymuses with ephrinB1 or ephrinB2 genes deleted in TECs, these changes begin at the perinatal stage. Mutant thymuses with ephrinB1 or ephrinB2 deleted in thymocytes, or with the ephrinB1 gene deleted or both ephrinB1/B2 genes deleted in TECs, contain a high number of large monolayer cysts formed by K5^+^K8^+^ cells and sometimes MTS-20^+^ cells, but rarely UEA-1^+^ or MTS-10^+^ cells. However, these mutants have a higher proportion of UEA-1^+^MTS-10^−^ cells, which often form small cysts surrounded by a thin rim of UEA-1^lo/−^MTS-10^+^, than WT ones.

These results raise several questions. Firstly, why do thymuses with defects in ephrinB1 and/or ephrinB2 contain numerous medullary epithelial cysts? The cell monolayer that constitutes the early thymic primordium acquires a 3D organisation when colonised by lymphoid progenitors. However, some polarised epithelial cells remain and form epithelial cysts [70]. Large, abundant monolayer cysts and small, dense medullary foci similar to those described in ephrinB-deficient thymuses [38] have been reported in the thymuses of mice undergoing an early blockade of T-cell differentiation [48,71,72,73]. Indeed, a reverse correlation has been emphasised between the presence of thymic cysts and the presence of thymocytes [74]. However, we were unable to establish any correlation between the number of thymocytes and cyst number or size. Instead, we speculate that the correct differentiation and organisation of the thymic epithelium, and the avoidance of excessive epithelial cysts, requires proper TEC and thymocyte disposition and physical contact through Eph/ephrinB interactions. Our results suggest that some mTEC populations are arranged concentrically around ‘central cores’ representing incipient medullary cysts. These TEC populations could include a progenitor cell subset that expresses UEA-1 and MTS-10, which appeared in high numbers in thymuses with ephrinB1 and ephrinB2 deleted in thymocytes [38].

On the other hand, the significance of this high number of medullary epithelial cysts for immune reactivity remains unclear. In other experimental models in which molecules involved in the maturation of the thymic epithelium are impaired, thymocyte differentiation is quite normal [14,36,43,44]. Similarly, ephrinB1- and/or ephrinB2-specific deletion in immature thymocytes results in a partial T-cell differentiation blockade at the DN3 cell stage [65], as previously reported by other authors studying similar mutant mice [33,75,76]. A lack of ephrinB1 in TECs is also associated with DN cell blockade, and double mutants deficient in ephrinB1/B2 genes exhibit a more severe phenotype characterised by a slight decrease in TCRαβ^+^ T cells and a reduction in TCRαβ^hi^CD69^+^ cells [65]. This suggests a role for Eph/ephrin in T-cell selection, as previously pointed out [33]. However, these results do not correlate with changes in the MHCII expression in mutant thymuses [65]. Furthermore, apart from decreased cell numbers, mutant lymphoid organs do not exhibit significant variations in the proportions of peripheral T lymphocytes. In agreement, Jin et al. [77] found no significant anomalies in mature T-cell function in mice with a conditional deletion of EphB4 in TECs.

In summary, the expression of ephrinB1 and ephrinB2 on thymocytes would be responsible for thymocyte–TEC interactions. The expression of ephrinB1 on TECs is necessary for the expansion, maturation and tridimensionalisation of mTECs, while the expression of ephrinB2 on TECs would be largely involved in determining the number of medullary foci (future epithelial cysts) and/or the distance between them. This is probably related to its involvement in TEC–TEC interactions [38].

### 3.3. The Reported Alterations in TEC Development in Eph- or Ephrin-Deficient Mice Do Not Block T-Cell Differentiation

It is assumed that proper T-cell differentiation requires an organised thymic epithelium. However, many experimental models exhibit a profoundly altered thymic microenvironment yet do not demonstrate significant alterations in thymocyte differentiation [36,43,44]. Studies on EphB- or ephrinB-deficient thymuses confirm this assumption.

As mentioned above, a lack of EphB or ephrinB results in a reduction in the number of both thymocytes [11,65] and TECs [13]. Apart from the reduced colonisation of the thymus by lymphoid progenitor cells that occurs in EphB2- and EphB3-deficient thymuses [23,25], hypocellularity correlates with increased apoptosis of DN and DP thymocytes. However, it is difficult to ascertain the relevance of the cell cycle in developing thymocytes because, in ephrinB1/B2-conditioned thymuses, there are increased proportions of cycling DP thymocytes [65], presumably in an attempt to restore normal thymic cell content [19]. Conversely, the absence of EphB2 and/or EphB3 resulted only in an increased proportion of total DN thymocytes and a reduced frequency of DN3 cells in adult thymuses [11].

Previously, we demonstrated that blocking EphA signalling using EphA1-Fc, EphA2-Fc, EphA3-Fc and ephrinA1-Fc fusion proteins resulted in significantly lower proportions of thymocytes, particularly both DN and DP thymocytes [10]. In vivo, EphA4-deficient mice also exhibit decreased proportions of DP cells and accumulation of DN3 thymocytes [68]. Meanwhile, murine FTOCs grown in the presence of ephrinB1-Fc fusion proteins yield decreased proportions of DP cells and increased proportions of SP cells [19].

### 3.4. The Disbalanced Eph/EphrinB Signalling Occurring in SCID-WT Mouse Chimaeras Clarifies the Role of Eph/Ephrin in T Cell Maturation

It is important to determine whether the changes reported in the T cell maturation of Eph/ephrin-deficient mice are due to a direct effect on the developing T cells, or whether they are a consequence of maturation in an altered thymic microenvironment. As both thymocytes and TECs express Eph and ephrins and responses to Eph/ephrin signalling result from the balance of total signals, we analysed the maturation of EphB2 and/or EphB3 or WT BM cell progenitors in SCID mice [78]. EphB2^−/−^ chimaeric thymuses showed a massive accumulation of DN cells, primarily DN2 and DN3 cells, and a near-total disappearance of DP thymocytes due to an increased proportion of apoptotic cells within this T cell subset. In contrast, EphB2LacZ thymuses exhibited a less severe phenotype than that of EphB2^−/−^ mice, suggesting the relevance of a reverse signal that could control DN–DP cell progression by providing survival signals from ephrinB-expressing cells to developing thymocytes. Thus, the regulation of thymocyte maturation by Eph/ephrins involves both autonomous controls, mediated by the developing thymocytes themselves, and non-autonomous controls involving TECs. EphB3 also appears to be involved in DN-to-DP cell progression, as EphB3^−/−^ chimaeras support the maturation of all T cell subsets, albeit with a significant reduction in the percentage of DP cells. The phenotype of chimaeras that are double-deficient in EphB2/B3 is quite similar to that of EphB2^−/−^ ones. However, the presence of EphB2 alone is insufficient to enable the normal progression to SP thymocytes; rather, both EphB2 and EphB3 are necessary for the final maturation of DP thymocytes into SP cells.

### 3.5. The Immune Condition of EphB-Deficient Mice

Despite the alterations to the thymic epithelial microenvironment and their impact on the thymocyte development, our pioneering studies revealed no significant effects on the immune capacities of mutant mice:

(a) The anatomy of the sacrificed mutants does not reveal any visual evidence of malignancy or significant pathological changes. The spleen and lymph nodes of the mutants are smaller in size, but have a normal histological organisation, which partly reflects the condition of the mutant thymuses [1].

(b) In fact, despite a significant reduction in cell content in the peripheral blood, mesenteric lymph nodes, and spleen of mutant mice, there are no significant differences in the proportions of peripheral CD4/CD8-defined T cell subsets in EphB-deficient mice [11].

(c) In mutant thymuses that are deficient in EphB2 and/or EphB3, there are no differences in the proportions of positively or negatively selected thymocytes, or in the proportions of total regulatory T (Treg) cells [79]. In the periphery, there are no changes in the percentages of Th1 cells, Th2 cells and Th17 cells. The proportion of Tregs remains unchanged in the spleen, but increases slightly in the inguinal lymph nodes of mutants compared to WT values [79].

(d) Previously, we analysed the TCRαβ repertoire of SP CD4^+^ cells in the thymus and lymph nodes of EphB-deficient and WT mice. Apart from some differences specific to each mutant, the only change observed in the three mutants was an increase in the proportion of Vβ3^+^CD4^+^ cells in both the thymus and lymph nodes [1].

In addition to the morphological and phenotypic changes that occur in the thymic epithelial microenvironment and/or the T-cell development in EphB- or ephrinB-deficient mice, it is important to establish whether these changes affect the immune capacities of the mutant animals. We recently examined these capacities in an experimental model of collagen-induced arthritis (CIA) generated in EphB2- and EphB3-deficient mice [80]. As previously reported, few changes occurred in the mutant thymic T-cell subpopulations known to be involved in autoimmunity compared to WT values. These include positively (TCRαβ^hi^CD4^+^CD8^−^CD69^+^ cells) and negatively selected T-cells (Caspase^+^CD4^+^CD8^−^CD5^+^CD69^+^ cells) and Treg subsets (TCRαβ^hi^Foxp3^+^ cells and TCRαβ^hi^Foxp3^+^CD4^+^ cells) [79].

Moreover, EphB2- and EphB3-deficient mice did not show lymphoid infiltrates identified on histological sections of the testes, salivary glands or adipose tissue [80]. Small lymphoid accumulations present in some liver sections of mutant mice were also observed in WT mice, as previously reported [81]. Furthermore, no reactivity to self-tissue antigens was detected in histological sections of WT salivary glands incubated with serum from either WT or EphB-deficient mice [80]. EphB-deficient mice respond scarcely (EphB3^−/−^ mice) or not at all (EphB2^−/−^ mice) to experimental arthritis induced by the subcutaneous injection of chicken type II collagen [80]. This condition has also been observed in models of rheumatoid arthritis or experimental autoimmune encephalomyelitis (EAE) induced in EphA4-deficient mice [82] or in mice lacking ephrinB1, ephrinB2 or double ephrinB1/B2, the main ligands of EphB2 and EphB3 [83,84,85].

Various factors may contribute to explaining all these results:

(1) The low immune reactivity observed in EphB2- and EphB3-deficient mice may be due to defects in central tolerance mechanisms as a consequence of T-cell maturation in an altered thymic microenvironment, despite the few changes observed in T-cell development. In EphB-deficient thymuses, proper thymic selection may occur if a sufficient number of thymocyte–TEC interactions occur, or if other antigen-presenting cells are present, such as dendritic cell subsets, B lymphocytes, or medullary fibroblasts, which are also involved in T-cell selection. However, according to our results, only the proportion of plasmacytoid dendritic cells increases in deficient thymuses [80], and the immune functions of these cells are a matter of discussion [86]. On the other hand, mTECs involved in the negative selection of self-reactive T cells in the thymus express transcripts that encode peripheral tissue antigens (PTAs) or tissue-specific antigens. Aire induces the expression of a large number of PTA transcripts [87], whereas the named thymic mimetic cells, described as “hybrid” cells of Aire^lo^Foxn1^lo^ mTECs and peripheral cell types, imitate peripheral cells in their chromatin landscape and transcriptomic profile and express tissue-specific antigens within the thymus [88]. Further studies have shown that these cells constitute a post-Aire thymic compartment characterised by the downregulation of Aire and MHCII expression. They include tuft cells, microfold cells, ciliated cells, keratinocytes, ionocytes, muscle cells, neuroendocrine cells, globet cells, basal cells (skin, lung), entero/hepatocytes and Ptf1a^+^ pancreatic mimetic cells. In fact, there is a significant degree of conversion of mimetic cell types across species, although some mimetic cells are species-specific [89]. Consequently, thymic mimetic cells represent a complementary mechanism to Aire-mediated PTAs expression in providing peripheral antigens to the thymic microenvironment. Nevertheless, evidence of the role of mimetic cells in human immunological tolerance is only indirect [88,90,91], and no published data exist on the status of these cell subsets in Eph- or ephrin-deficient thymuses, an issue that will be addressed next.

(2) The limited reactivity may also be a consequence of the condition of the peripheral T cells, which are directly responsible for arthritic lesions. Defects in EphB6 are associated with compromised lymphokine secretion, T cell proliferation and delayed-type skin hypersensitivity, despite normal proportions of thymic T cell subsets [17]. In other models, ameliorated development of EAE [83] and CIA in ephrinB1/B2 double KO mice was associated with defective Th1 and Th17 cells [83]. Together with our own results, these data suggest that signalling via EphB2, EphB3, ephrinB1 and ephrinB2 is necessary for an adequate immune response.

In our model, the draining inguinal lymph nodes of non-immunised mice exposed to chicken type II collagen exhibit a tendency to produce fewer reactive T cells and higher proportions of Treg cells in EphB3^−/−^ mice than in non-immunised WT mice. There are also increased percentages of IL4-producing cells in both EphB2- and EphB3-KO mice. IL4 appears to play a key role in determining the occurrence (or not) of CIA, as observed in other studies [92], and its administration reduces clinical parameters [93,94] and bone erosion and joint damage [95]. EphB2^−/−^ mice exhibit reduced reactivity to collagen stimulation, with low antibody production and decreased proliferation of inguinal lymph node T cells in response to anti-CD3 and anti-CD28 antibody stimulation. On the other hand, EphB3^−/−^ mice also show decreased reactivity. In particular, inguinal lymph node T lymphocytes exhibit minimal capacity to migrate to CXCL12 in transwell assays [80].

These results suggest that EphB-deficient mice respond apparently well immunologically as evidenced by the absence of autoreactive lymphoid infiltrates and autoantibodies. However, alterations in the functional properties of peripheral T cells, affecting either antigen reactivity or cell migration to inflamed tissues, hinder the progression of arthritis.

### 3.6. The Phenotype of EphA4^−/−^ Thymuses Reinforces the Specificity of Eph/Ephrin-Mediated Effects and the Role of These Molecules in Thymocyte–TEC Interactions

For a more detailed understanding of the relevance of Eph/ephrin signalling to thymus biology, we compared the results reported on EphB- or ephrinB-deficient thymuses with those obtained by studying EphA4^−/−^ mice [68,96]. The analysis of the thymic phenotypes exhibited by EphA4^−/−^ mice supports the aforementioned distinct specificity of Ephs and ephrins in families A and B for determining thymus structure and function. However, more importantly, it provides remarkable information on the relevance of Eph/ephrin-mediated thymocyte–TEC interactions in controlling thymus biology.

In contrast to the EphB- or ephrinB-deficient thymuses, which showed few changes in T-cell maturation [11], data on EphA4^−/−^ thymuses showed an early blockade of T-cell progenitor differentiation. This resulted in the accumulation of DN3 thymocytes. Notably, this altered T-cell differentiation occurred in thymuses with an extremely reduced, collapsed cortex. Additionally, WT BM lymphoid precursor cells undergo profoundly altered T-cell development in the presence of EphA4-deficient thymic stroma [68].

More recently, we classified EphA4^−/−^ thymuses according to their DP thymocyte content [96]. Interestingly, some mutant mice contained a high proportion of DP cells (DP^hi^ thymuses), which was quite similar to that of WT thymuses. Others showed low proportions of DP thymocytes (DP^lo^ thymuses). In the intermediate condition (DP^Int^ thymuses), the DP cell compartment contained 50–70% of DP thymocytes.

The thymuses of EphA4^−/−^ mice, which contain high proportions of DP thymocytes, did not exhibit changes in the percentages of DN cells, but showed reduced proportions of positively selected thymocytes and Treg cells, with no changes observed in the proportions of the CD69^−^CD62L^+^ cell subset. Together, these findings could explain the decrease in SP thymocytes observed in these DP^hi^ mutants. EphA4^−/−^ thymuses containing low proportions of DP cells (DP^lo^ thymuses) were of particular interest. These showed increased proportions of DN3 cells and low numbers of DN4 thymocytes. Both DP^lo^ and DP^Int^ thymuses contained increased proportions of positively selected thymocytes and Treg cells, with no changes observed in the proportions of negatively selected T cells. These results, together with the accumulation of mature CD69^−^CD62L^+^ CD4^+^/CD8^+^ cells, contributed to an increased percentage of SP cells in these deficient thymuses [96]. However, there is no evidence of a direct relationship between EphA4/ephrin signalling and thymic selection. Nevertheless, EphA4 KO mice show a less severe autoimmune response when EAE is induced [82]. Furthermore, Krox20/Erg2B, a transcription factor involved in thymocyte selection [97], regulates EphA4 expression, and ephrinA-Fc fusion proteins and anti-CD3 monoclonal antibodies suppress CD69 expression [20].

More importantly, changes in T-cell maturation in EphA4-deficient mice correlate well with significant alterations to the thymic epithelium [68,96]. Histologically, collapse of the thymic cortex epithelium (Figure 1), particularly in DP^lo^ thymuses, is associated with an increased proportion of Ly51^+^UEA-1^−^ cTECs and a reduced proportion of Ly51^−^UEA-1^+^ mTECs, possibly due to defective maturation of MHCII^lo^CD80^−^ immature mTECs. The increased proportions of EphA4^−/−^ cTECs obliterate empty cortical areas due to the lack of DP thymocytes, resulting in the collapse of the thymic cortex. Meanwhile, enlargement of the thymic medulla, despite reduced numbers of mature mTECs, is due to increased proportions of positively selected SP thymocytes and Treg cells alongside unchanged negative selection [96]. There are indeed changes in ECM components (collagen type IV, FN, LN) and their receptors (VLA-4, VLA-6), which affect cell adhesion rather than thymocyte migration mediated by chemokines (CXCL12; CCL25, CCL21) and their receptors (CXCR4, CCR7, CCR9) [96].

## 4. Could Mice with a Profoundly Altered Thymic Stroma Still Be Immunocompetent?

As mentioned above, numerous examples in the scientific literature show that alterations to the thymic epithelium, which provoke a total or partial lack of thymocyte–TEC interactions due to distinct processes, molecules, or signalling pathways, do not necessarily result in changes to the phenotype of T cell subsets or in a breakdown of central tolerance [36,44,77,98,99,100]. In other words, some authors have suggested that only a small number of TECs would be enough to facilitate the necessary interactions between thymocytes and TECs to support the maturation of lymphoid cells [36].

In this respect, all of our studies on Eph/ephrins and the organisation of the thymus lead to one clear conclusion: interactions between thymocytes and TECs are essential for proper immunological functioning of the organ [79]. However, could an apparently altered thymic microenvironment still support key thymic functions, such as central immunological education? In other words, are mice with a profoundly altered thymic microenvironment immunocompetent? To test this hypothesis, we studied T-cell maturation in RTOCs containing varying numbers of E14.5 thymic stromal cells (TSCs). One month after grafting under the kidney capsule of Foxn1^−/−^ mice [79], RTOCs established with 1 − 0.5 × 10^6^ WT TSCs had grown around 5–7 times, whereas those containing fewer TSCs (0.085 × 10^6^ WT TSCs) grew significantly less and exhibited a reduced proportion of DP thymocytes and increased frequencies of TCRαβ^hi^ cells.

## 5. Conclusions and Perspectives

These results all demonstrate the important role of the Eph receptor family and its ligands, the ephrins, in organising the thymic epithelial stroma and thymocyte development in mice. The different functions of the various members of these families of molecules support the specificity of the process.

The absence of EphB2 and/or EphB3, or ephrins B, results in significant changes to the thymic epithelial stroma. However, this does not prevent T-cell differentiation, which shows only minor alterations in the thymus and peripheral lymphoid organs.

By contrast, the absence of EphA4 signalling blocks T-cell differentiation, resulting in varying proportions of DP thymocytes and a histological collapse of the thymic epithelial cortex.

These results partially question the necessity of correct histological organisation of the thymic stroma in order to support proper T cell maturation, or at least to preserve certain aspects of the process.

On the other hand, although many questions remain about the role of different Ephs and ephrins in organising the lymphoid environment and T-cell development and function, three issues seem particularly important for further analysis:

(a) The condition of the thymic medullary mimetic cells in Eph- or ephrin-deficient mice must be examined. Alterations to these cells, which are involved in the intrathymic presentation of PTAs, could help to explain certain defects in thymic education.

(b) The immune capacities of thymocytes and peripheral T lymphocytes in mutant mice with different defects in Eph or ephrin family molecules, beyond changes in their proportions or phenotypes, should be examined. 

(c) There is a possibility that a sufficient number of TEC–thymocyte interactions allows proper immune functioning or partially preserves certain capabilities independently of the histological organisation of the thymic epithelium. The results we have reported, which were obtained using in vivo grafted RTOCs formed with a varying number of WT TSCs, need to be confirmed by studying the behaviour of RTOCs generated with EphB-deficient thymic stromal cells.

## Data Availability

No new data were created or analyzed in this study. Data sharing is not applicable to this article.

## Abbreviations

The following abbreviations are used in this manuscript:

Abbreviation	Full Term
3D	Three-dimensional
BM	Bone Marrow
CIA	CIA Collagen-Induced Arthritis
cTEC	Cortical Thymic Epithelial Cell
DN	Double-Negative
DP	Double-Positive
E	Embryonic day
EAE	Experimental Autoimmune Encephalomyelitis
ECM	Extracellular Matrix
EFA	Epithelial-Free Area
Eph	Erythropoietin-Producing Hepatocellular Carcinoma
Ephrin	Eph Receptor-Interacting Protein
FN	Fibronectin
FTOC	Foetal Thymus Organ Culture
KO	Knock Out
LN	Laminin
MHC	Major Histocompatibility Complex
mTEC	Medullary Thymic Epithelial Cell
PTA	Peripheral Tissue Antigen
RTOC	Reaggregate Thymus Organ Culture
SP	Single Positive
TEC	Thymic Epithelial Cell
Treg	Regulatory T cell
TSC	Thymic Stromal Cell
WT	Wild-Type
